# Resectability and Neoadjuvant Chemoimmunotherapy in Stage II-III Non-Small Cell Lung Cancer: A European Case-Vignette Survey

**DOI:** 10.1093/icvts/ivag196

**Published:** 2026-07-07

**Authors:** Savvas Lampridis, Akshay J Patel, Eleni Josephides, Fabian Doerr, Carmelina Cristina Zirafa, Seray Hazer, Andres Obeso, Ilies Bouabdallah, Monica Casiraghi, Andrea Billè

**Affiliations:** Department of Thoracic Surgery, 424 General Military Hospital, Thessaloniki, Greece; National Heart and Lung Institute, Faculty of Medicine, Imperial College London, London W12 0NN, United Kingdom; Department of Thoracic Surgery, Guy’s Hospital, London SE1 9RT, United Kingdom; Institute of Immunology and Immunotherapy, University of Birmingham, Birmingham B152TT, United Kingdom; Department of Radiation Oncology, Guy’s Hospital, London SE19RT, United Kingdom; Department of Thoracic Surgery, West German Cancer Center, University Hospital Essen-Ruhrlandklinik, Essen, Germany; National Center for Tumor Diseases (NCT) West, Essen, Germany; Department of Thoracic Surgery, University Hospital of Pisa, Pisa, Italy; Department of Thoracic Surgery, Ankara Atatürk Sanatorium Training and Research Hospital, Ankara, Turkey; Heart & Vascular Institute, Cleveland Clinic Abu Dhabi, Abu Dhabi, United Arab Emirates; Department of Thoracic Surgery, Saint Joseph Hospital, Marseille, Provence-Alpes-Côte d’Azur, France; Department of Thoracic Surgery, IEO-European Institute of Oncology IRCCS, Milan, Italy; Department of Oncology and Hemato-Oncology, University of Milan, Milan, Italy; Department of Thoracic Surgery, Guy’s Hospital, London SE1 9RT, United Kingdom; School of Cancer and Pharmaceutical Sciences, King’s College London, London SE19RT, United Kingdom

**Keywords:** non-small cell lung cancer, resectability, neoadjuvant chemoimmunotherapy, multimodal therapy, case vignette survey

## Abstract

**Objectives:**

Immunotherapy has expanded curative-intent options in non-small cell lung cancer (NSCLC), but management recommendations remain variable. This survey examined variation in resectability assessment and treatment sequencing among thoracic oncology clinicians managing stage II-III NSCLC.

**Methods:**

A web-based case-vignette survey was distributed to thoracic oncology clinicians, primarily in Europe. Seven stage II-III NSCLC vignettes, based on the ninth-edition TNM classification, were presented. Treatment options were recorded as independent yes/no endorsements. The primary outcome was endorsement of neoadjuvant chemoimmunotherapy among vignette evaluations considered resectable. Vignette-level binary outcomes were analysed using exploratory generalized estimating equations clustered by respondent.

**Results:**

Sixty-eight respondents provided vignette-level assessments. Resectability was generally endorsed in the N0/N1 and selected single-station N2 scenarios but was substantially lower in the multi-station and T3/T4 N2 scenarios. Among cases considered resectable, endorsement of neoadjuvant chemoimmunotherapy increased markedly in N2 scenarios, whereas cases considered unresectable were commonly associated with endorsement of concurrent chemoradiotherapy. In an exploratory model restricted to resectable-branch evaluations (249 observations from 57 respondents), any N2 involvement was associated with endorsement of neoadjuvant chemoimmunotherapy (adjusted odds ratio 28.42, 95% CI 7.31-110.42; *P *< .001), though the wide interval reflects the limited effective sample.

**Conclusions:**

Variation was greatest in borderline N2 disease, particularly multi-station and higher-T N2 scenarios, where clinicians diverged in defining resectability and in selecting surgery-based vs chemoradiotherapy-based pathways. These findings support the need for clearer multidisciplinary frameworks in stage II-III NSCLC, within the limits of a surgeon-weighted respondent pool and an anatomically focused vignette set.

## Graphical abstract

**Figure:**
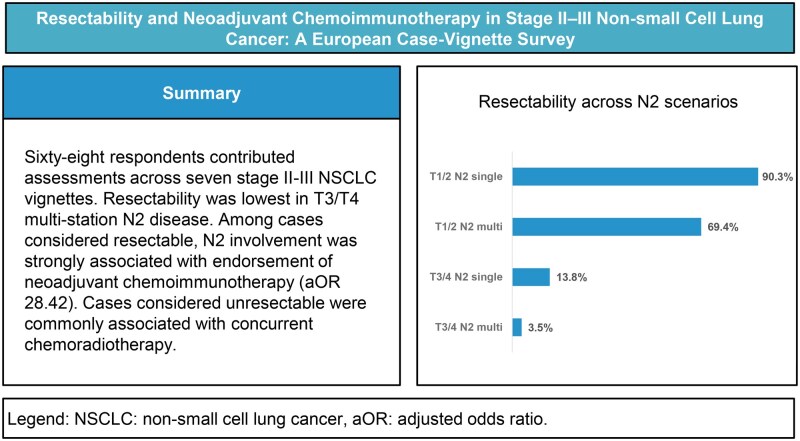

## INTRODUCTION

Lung cancer remains the leading cause of cancer mortality worldwide, with non-small cell lung cancer (NSCLC) accounting for approximately 85% of cases.^1^^,^^2^ For resectable stage II-III NSCLC, surgery with adjuvant platinum-based chemotherapy has long been the standard of care.^3^ However, more than half of these patients relapse within 5 years of complete resection, which reflects the limited disease control provided by this strategy.^4^

Immune-checkpoint inhibitors that target the PD-1/PD-L1 pathway have transformed the treatment of metastatic NSCLC and prompted extensive investigation of their role in earlier-stage disease. Indeed, multiple phase II and III trials have now demonstrated that integrating immunotherapy with platinum-based chemotherapy before or around surgery significantly improves pathological and survival outcomes compared with chemotherapy alone.^5–11^ Meta-analyses have confirmed consistent improvements in major and complete pathological response, event-free survival, and overall survival across histological and PD-L1 subgroups.^12–14^ In parallel, for unresectable stage III NSCLC, concurrent chemoradiotherapy followed by consolidation immunotherapy has become an established curative-intent strategy.^15–17^ These developments have informed recent guideline updates from the European Society for Medical Oncology and the National Institute for Health and Care Excellence, as well as consensus recommendations from the International Association for the Study of Lung Cancer.^18–20^

Despite these advances, real-world implementation remains heterogeneous. In particular, definitions of resectability and the criteria used to frame curative-intent management for stage II-III NSCLC vary across centres and health systems.^21–23^ However, contemporary evidence on how clinicians make these decisions in the immunotherapy era remains limited. We, therefore, conducted a predominantly European case-vignette survey to examine how thoracic oncology clinicians judge resectability and endorse management options for stage II-III NSCLC. Our aim was to map areas of agreement and divergence in contemporary practice, including exploratory specialty- and region-related patterns, and to identify where more consistent multidisciplinary frameworks may be needed.

## METHODS

### Study design

We conducted a cross-sectional, web-based case-vignette survey examining resectability judgements and management recommendations for stage II-III NSCLC. The study adhered to the Consensus-Based Checklist for Reporting of Survey Studies.^24^ The survey was hosted on the REDCap platform of Guy’s and St Thomas’ NHS Foundation Trust (London, United Kingdom) and remained open from September 4, 2024 to June 1, 2025. Participation was anonymous and voluntary, and no incentives were offered. Each participant received an individualized REDCap survey link, which prevented duplicate submissions. Before survey entry, participants were shown an information page describing the study purpose, confidentiality safeguards, and data-protection arrangements; proceeding to the questionnaire was taken as informed consent. Data were stored on secure institutional servers and managed in accordance with the General Data Protection Regulation. The institutional review board classified the project as exempt because it involved anonymized clinician responses only.

### Participants

The survey targeted thoracic surgeons, oncologists, and respiratory physicians involved in the multidisciplinary management of NSCLC. Invitations were disseminated through professional networks and mailing lists of the European Association for Cardio-Thoracic Surgery, the European Society of Thoracic Surgeons, and the British Thoracic Oncology Group, supplemented by institutional contacts.

### Survey instrument

The questionnaire included respondent demographics and 7 standardized clinical vignettes based on anonymized real cases and mapped to the ninth-edition TNM classification.^25^ Each vignette provided the intended tumour and nodal stage, the involved nodal stations, and representative staging imaging. By design, the vignettes did not include PD-L1 status, actionable driver alterations, or detailed physiological operability variables, because the instrument was intended to capture resectability judgements and broad curative-intent management recommendations rather than biomarker-directed treatment selection or full physiological operability assessment. Respondents were not provided with a prescribed definition of resectability; this was a deliberate design choice, as variation in how individual clinicians define resectability, including the relative weighting of technical and oncological considerations, was itself the object of study. Full vignette texts and imaging are provided in the **Supplementary Material**.

The survey followed a fixed branching structure for each vignette. Respondents were first asked whether the case was resectable (yes/no). If a vignette was judged resectable, respondents were shown a resectable branch comprising separate yes/no items on upfront surgery, neoadjuvant chemoimmunotherapy, and perioperative immunotherapy, together with a performance-status threshold item for surgery or induction treatment. If a vignette was judged unresectable, respondents were shown an unresectable branch comprising a yes/no item on concurrent chemoradiotherapy. If concurrent chemoradiotherapy was endorsed, respondents could then answer additional items on the minimum performance status acceptable for this approach and on consolidation immunotherapy after chemoradiotherapy. If concurrent chemoradiotherapy was not endorsed, a free-text reason could be entered. All items were optional, and treatment items were recorded as independent yes/no endorsements.

The survey instrument was developed by a multidisciplinary team of thoracic surgeons and oncologists with extensive experience in NSCLC management. Draft items were reviewed by external specialists to ensure clarity and content validity. Pilot testing with 5 clinicians confirmed acceptability and survey flow.

### Outcomes

The primary inferential outcome was endorsement of neoadjuvant chemoimmunotherapy, recorded as a binary yes/no variable at the respondent-vignette level. Because this item was displayed only after a vignette had first been judged resectable, the primary outcome was observable only within the resectable branch. Accordingly, the multivariable analysis was restricted to vignette evaluations considered resectable.

Secondary descriptive outcomes included resectability judgement, endorsement of upfront surgery, perioperative immunotherapy, concurrent chemoradiotherapy, and consolidation immunotherapy after chemoradiotherapy, together with the reported minimum performance-status thresholds for surgery or induction treatment and for concurrent chemoradiotherapy. Resectability was analysed descriptively because it was inherently vignette-specific and preceded branch allocation within the instrument.

### Data management

The unit of analysis was the respondent-vignette observation, with each respondent able to contribute up to 7 vignette evaluations. We defined analytic populations a priori at both the respondent and respondent-vignette levels. Because the survey used conditional branching and optional items, the number of respondents and observations contributing to each analysis could differ across outcomes.

Country entries were harmonized for spelling variants and grouped as Europe vs outside Europe. Clinician role was grouped a priori as thoracic surgeon vs non-surgeon. Respondents who did not report role or country were retained for descriptive analyses when outcome data were available but were excluded from regression models requiring those covariates.

Not all unobserved vignette-level items represented missing data. We distinguished 3 forms of non-observation: structural inapplicability, attrition, and true item non-response. Structural inapplicability referred to items not displayed because the respondent entered the opposite survey branch; attrition referred to vignette pages not reached; and true item non-response referred to eligible items left unanswered after the relevant branch had been entered. Missing data were not imputed.

### Statistical analysis

Categorical variables were summarized as counts and percentages with Wilson 95% CIs, and continuous variables as medians with interquartile ranges (IQR). Descriptive analyses were outcome-specific and used the number of observed responses for the relevant item as the denominator.

The primary inferential analysis examined endorsement of neoadjuvant chemoimmunotherapy at the respondent-vignette level using a generalized estimating-equations logistic model with an exchangeable working correlation matrix and clustering by respondent. Because the neoadjuvant chemoimmunotherapy item was displayed only after a vignette had been judged resectable, this model was restricted to vignette evaluations within the resectable branch in which the primary outcome was observed. The primary predictor was nodal status, dichotomized as any N2 vs N0/N1. Clinician specialty (thoracic surgeon vs non-surgeon) and practice region (Europe vs outside Europe) were included a priori as covariates. Adjusted odds ratios (aOR) were estimated with robust standard errors, 95% CI, and Wald *P* value. Model-based predicted probabilities were derived by marginal standardization.

Only respondent-vignette observations with an observed primary outcome and non-missing covariate data were included in the multivariable model. At the respondent level, the proportion of answered eligible vignettes for which neoadjuvant chemoimmunotherapy was endorsed was compared between thoracic surgeons and non-surgeons using the Wilcoxon rank-sum test. All tests were 2-sided with *α* = 0.05. Analyses were performed using SPSS Statistics version 29.0 (IBM Corp., Armonk, NY, United States).

## RESULTS

**Figure 1.**
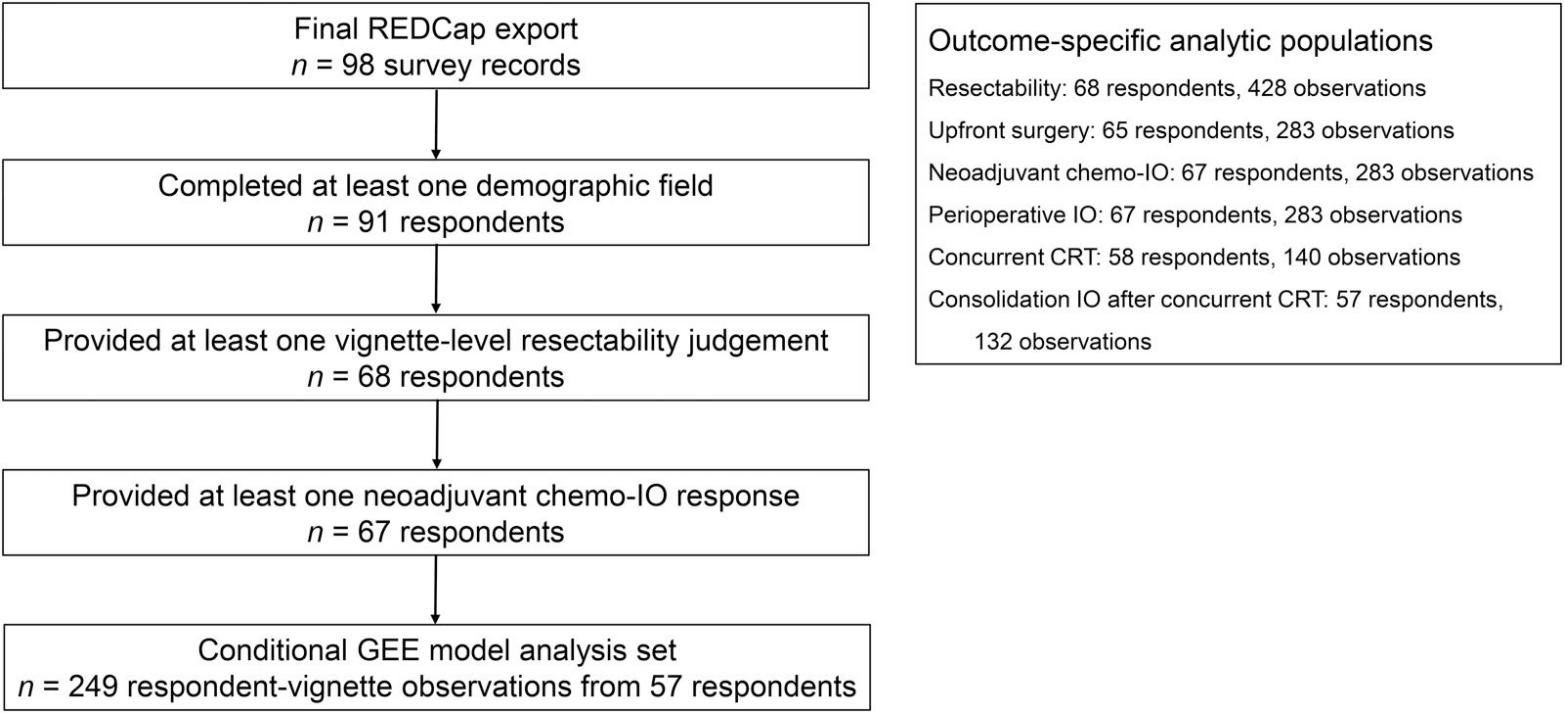
Respondent Flow and Analysis Sets Abbreviations: chemo-IO, chemoimmunotherapy; CRT, chemoradiotherapy; GEE, generalized estimating equations; IO, Immunotherapy.

The final REDCap export contained 98 survey records, of which 91 respondents completed at least one demographic field. Respondent characteristics are summarized in **Table 1**. Sixty-eight respondents provided at least one vignette-level resectability judgement, and 67 provided at least one neoadjuvant chemoimmunotherapy response. The respondent flow, outcome-specific analysis sets, and conditional multivariable-model dataset are shown in **Figure 1**. Detailed outcome-specific analytic populations and branch accounting for the primary outcome are provided in **Table S1** and **Table S2**, respectively.

**Table 1.** Respondent Characteristics

Characteristic	*n*/*N* (%)
Specialty	
Thoracic surgeon	77/91 (84.6)
Oncologist	7/91 (7.7)
Respiratory physician	3/91 (3.3)
Other	4/91 (4.4)
Region	
United Kingdom	22/71 (31.0)
Other Europe	32/71 (45.1)
Outside Europe	17/71 (23.9)
Recruitment source	
EACTS	36/42 (85.7)
ESTS	5/42 (11.9)
BTOG	1/42 (2.4)

Percentages are based on non-missing responses for each variable.

Abbreviations: BTOG, British Thoracic Oncology Group; EACTS, European Association for Cardio-Thoracic Surgery; ESTS, European Society of Thoracic Surgeons.

Across the ordered vignette set, respondent reach declined modestly, and resectability judgements varied substantially by scenario. Resectability was generally endorsed in N0/N1 disease and in selected single-station N2 scenarios, but it was less frequently supported in multi-station N2 involvement and higher T categories. Full vignette-level data on resectability and resectable-branch endorsements are presented in **Table 2**, and unresectable-branch results are shown in **Table 3**.

**Table 2.** Resectability and Resectable-Branch Endorsements

Vignette no. and label	Resectable	Upfront surgery	Neoadjuvant chemoimmunotherapy	Perioperative immunotherapy
1. T3 (>5 cm) N0	68/68 (100)	45/65 (69.2)	32/67 (47.8)	38/67 (56.7)
2. T1/2 N1 (11 l)	63/64 (98.4)	31/62 (50.0)	38/62 (61.3)	49/62 (79.0)
3. T1/2 N2 single station (4 R)	56/62 (90.3)	8/56 (14.3)	53/55 (96.4)	50/55 (90.9)
4. T1/2 N2 multi-station (4 R+7)	43/62 (69.4)	2/43 (4.7)	43/43 (100)	42/42 (100)
5. T3/4 N1 (11 R)	47/57 (82.5)	11/47 (23.4)	39/46 (84.8)	41/47 (87.2)
6. T3/4 N2 single station (4 l)	8/58 (13.8)	0/8 (0.0)	8/8 (100)	8/8 (100)
7. T3/4 N2 multi-station (2 R+4R)	2/57 (3.5)	0/2 (0.0)	2/2 (100)	2/2 (100)

Values are *n*/*N* (%). Resectable percentages use respondents who reached that vignette as the denominator. Resectable-branch treatment items use eligible observations with a recorded response. Treatment items were independent yes/no endorsements and were not mutually exclusive.

**Table 3.** Unresectable-Branch Endorsements

Vignette no. and label	Unresectable	Concurrent chemoradiotherapy	Consolidation immunotherapy after concurrent chemoradiotherapy
1. T3 (>5 cm) N0	0/68 (0.0)	—	—
2. T1/2 N1 (11 l)	1/64 (1.6)	1/1 (100)	0/1 (0.0)
3. T1/2 N2 single station (4 R)	6/62 (9.7)	6/6 (100)	5/6 (83.3)
4. T1/2 N2 multi-station (4 R+7)	19/62 (30.6)	19/19 (100)	19/19 (100)
5. T3/4 N1 (11 R)	10/57 (17.5)	9/10 (90.0)	9/9 (100)
6. T3/4 N2 single station (4 l)	50/58 (86.2)	47/50 (94.0)	45/46 (97.8)
7. T3/4 N2 multi-station (2 R+4R)	55/57 (96.5)	51/54 (94.4)	49/51 (96.1)

Values are *n*/*N* (%). Unresectable evaluations are defined by a negative resectability judgement. Branch-specific treatment items use eligible observations with a recorded response. A dash indicates no eligible observations.

Within the resectable branch, endorsement of upfront surgery was most frequent in the earlier-stage scenarios and became uncommon in the more advanced cases. By contrast, endorsement of neoadjuvant chemoimmunotherapy increased markedly in the N2 scenarios that were still considered resectable (**Table 2**). Upfront surgery and neoadjuvant chemoimmunotherapy were not mutually exclusive; the co-endorsement pattern is shown in **Table S3**.

Within the unresectable branch, concurrent chemoradiotherapy was endorsed in most eligible observations across the more advanced scenarios. Once concurrent chemoradiotherapy had been endorsed, consolidation immunotherapy was also recommended in most follow-up observations (**Table 3**). Reported Karnofsky performance-status thresholds for offering surgery or neoadjuvant chemoimmunotherapy were generally centred around 60 across scenarios; corresponding thresholds for concurrent chemoradiotherapy are shown in **Table S4**.

Specialty-related patterns warrant cautious interpretation given the small non-surgeon subgroup. Thoracic surgeons more often endorsed resectability and upfront surgery, whereas non-surgeons more often endorsed neoadjuvant chemoimmunotherapy within eligible resectable-branch observations. At the respondent level, the median proportion of answered eligible vignettes endorsed for neoadjuvant chemoimmunotherapy was 100% (IQR 100-100) among non-surgeons and 80% (IQR 50-100) among thoracic surgeons (Wilcoxon rank-sum *P *= .054). Regional differences were less marked.

In the exploratory conditional generalized estimating-equations model, 249 resectable-case observations from 57 respondents were analysed (**Table 4**). Within this conditional analysis set, any N2 involvement was associated with endorsement of neoadjuvant chemoimmunotherapy compared with N0/N1 disease (aOR 28.42, 95% CI 7.31-110.42; *P *< .001). Non-surgeon specialty was also associated with greater endorsement of neoadjuvant chemoimmunotherapy than thoracic-surgeon specialty (aOR 8.05, 95% CI 1.20-54.07; *P *= .032), whereas region was not associated with the outcome (Europe vs outside Europe: aOR 1.46, 95% CI 0.34-6.22; *P *= .606).

**Table 4.** Conditional Generalized Estimating-Equations Logistic Model for Endorsement of Neoadjuvant Chemoimmunotherapy

Comparison	Adjusted OR	95% CI	*P* value
Any N2 vs N0/N1	28.42	7.31-110.42	<.001
Non-surgeon vs thoracic surgeon	8.05	1.20-54.07	.032
Europe vs outside Europe	1.46	0.34-6.22	.606

Outcome: endorsement of neoadjuvant chemoimmunotherapy. Analysis set: 249 respondent-vignette observations from 57 respondents. The outcome was observed only in the resectable branch.

Abbreviations: CI, confidence interval; OR, odds ratio.

## DISCUSSION

This vignette-based survey provides a structured map of practice variation in stage II-III NSCLC under standardized anatomical scenarios. Two patterns were most apparent. First, variation emerged at the level of resectability assessment, particularly in the intermediate and more advanced N2 scenarios. Second, among cases judged resectable, endorsement of neoadjuvant chemoimmunotherapy increased markedly in N2 disease, whereas more advanced scenarios more often entered the unresectable branch and were commonly associated with endorsement of concurrent chemoradiotherapy.

The resectability patterns observed here are broadly consistent with the continuing uncertainty surrounding stage III NSCLC. In the recent European Organization for Research and Treatment of Cancer (EORTC) survey for defining resectable stage III NSCLC for clinical trials, panelists reached agreement mainly at the extremes: N3 involvement, bulky or invasive N2 nodes, and T4 tumours with multi-station N2 disease were considered unresectable, whereas a range of multi-station and higher-T single-station N2 configurations remained unsettled.^26^ Using the EORTC’s 75% agreement threshold as a benchmark, 4 of our 7 vignettes would be classified as resectable (T3N0, T1/2N1, T1/2N2 single-station, and T3/4N1), while 2 would be classified as unresectable (T3/4N2 single- and multi-station, respectively). Only one configuration, T1/2N2 multi-station, occupies an intermediate zone, with approximately 69% of clinicians judging it resectable. This pattern mirrors the EORTC survey findings and supports the view that, even in the immunotherapy era, clinicians do not apply TNM descriptors in isolation: resectability has both a technical and an oncological dimension, and disagreement in borderline N2 disease often reflects different weightings of these 2 components.

Within the resectable branch, endorsement of neoadjuvant chemoimmunotherapy rose sharply in N2 scenarios, while upfront surgery was more often endorsed in the less advanced cases. In the unresectable branch, concurrent chemoradiotherapy was endorsed in most eligible observations, often followed by consolidation immunotherapy. These findings are directionally consistent with current multimodal treatment frameworks for resectable and unresectable locally advanced NSCLC.^18–20^

The exploratory conditional model should be interpreted narrowly. Because the neoadjuvant chemoimmunotherapy item was observable only within the resectable branch, the model quantifies one pattern within resectable-case evaluations rather than a general model of treatment allocation, and the wide CI (7.31-110.42) reflects the limited effective sample size. Within that restricted analysis set, N2 disease was associated with endorsement of neoadjuvant chemoimmunotherapy, a finding consistent with the descriptive data. The study’s contribution, therefore, lies in mapping where variation persists under standardized anatomical scenarios, not in demonstrating an N2–chemoimmunotherapy association that is independently expected from current evidence.

Specialty-related differences were also observed, with non-surgeons more likely to endorse neoadjuvant chemoimmunotherapy and surgeons more likely to endorse resectability and upfront surgery. Similar specialty-related patterns have been reported in other case-based studies of stage III NSCLC. In a Swiss survey of N2 disease, surgeons frequently recommended surgery-based strategies for non-bulky or even multilevel N2 involvement, whereas radiation oncologists increasingly favoured radiotherapy-based approaches as mediastinal extent increased.^27^ Comparable specialty effects were observed in another survey of stage III NSCLC recommendations, in which surgeons were more likely than medical oncologists to recommend surgical management across a range of stage III scenarios, despite chemoradiotherapy remaining the predominant overall recommendation.^28^ However, the small non-surgeon subgroup in our survey limits precision, and these specialty-related patterns should be read as signals rather than as conclusions about how different disciplines would behave in broader practice.

A strength of the vignette design is that it standardizes the anatomical information presented to respondents and thereby allows structured comparison of how clinicians interpret similar scenarios. At the same time, such a design cannot reproduce the full complexity of multidisciplinary decision-making: it cannot capture institutional experience, local access pathways, or the iterative discussion through which final recommendations are often reached. The vignette format, therefore, complements, rather than substitutes for, observational studies of delivered care.

Several limitations should be acknowledged. First, the branching structure of the survey means that not all items were available for all vignette evaluations, and the primary outcome was observable only after a resectable judgement. Second, treatment endorsements were not mutually exclusive, which appropriately reflects uncertainty and overlap in real practice but limits interpretation as a measure of single-pathway treatment selection. Third, respondents were not given a prescribed definition of resectability; this was a deliberate design feature, since variation in how clinicians define resectability was itself, the object of study, but it means that observed variation reflects both differences in judgement and differences in operative definitions. Fourth, the respondent pool was predominantly European and surgeon-weighted, and subgroup analyses were based on small numbers. Fifth, although representative staging imaging was provided, the vignettes did not include biomarker data or the full clinical and physiological detail that often determines real-world operability and treatment sequencing; these omissions are deliberate features of the instrument’s anatomical focus rather than oversights, but they constrain extrapolation to fully biomarker- and physiology-stratified multidisciplinary practice. Finally, respondent reach declined modestly across the ordered vignette set, so some later-case denominators were reduced by survey attrition in addition to branch eligibility.

Future work should focus on refining and standardizing definitions of resectability in stage III NSCLC and on linking multidisciplinary assessments with delivered treatment and clinical outcomes in prospective datasets. Such studies may help determine how variation observed in standardized vignette-based scenarios translates into real-world practice.

## CONCLUSION

In this predominantly European case-vignette survey, variation in stage II-III NSCLC management was greatest in borderline N2 disease, particularly multi-station and higher-T N2 scenarios, where clinicians diverged both in resectability judgement and in the choice between surgery-based and chemoradiotherapy-based pathways. These findings support the need for clearer multidisciplinary frameworks and careful case-by-case assessment when applying contemporary multimodal strategies.

## Supplementary Material

ivag196_Supplementary_Data

## Data Availability

The data underlying this study are available from the corresponding author on reasonable request.
