# Supplementary material for: Resectability and Neoadjuvant Chemoimmunotherapy in Stage II-III Non-Small Cell Lung Cancer: A European Case-Vignette Survey
Source: Interdiscip Cardiovasc Thorac Surg. 2026 Jul 7;41(7):ivag196. doi: 10.1093/icvts/ivag196 (PMC13385344; doi:10.1093/icvts/ivag196)
Supplement: ivag196_Supplementary_Data [file ivag196_supplementary_data.zip › Supplementary Material 2.docx]

**Table S1.** Outcome-specific Analytic Populations

| **Outcome** | **Respondents with at least one observed response, *n*** | **Respondent-vignette observations, *n*** | **Eligibility rule** |
| --- | --- | --- | --- |
| Resectability | 68 | 428 | Asked first for each vignette reached |
| Upfront surgery | 65 | 283 | Asked only within the resectable branch |
| Neoadjuvant chemoimmunotherapy | 67 | 283 | Asked only within the resectable branch |
| Perioperative immunotherapy | 67 | 283 | Asked only within the resectable branch |
| Concurrent chemoradiotherapy | 58 | 140 | Asked only within the unresectable branch |
| Consolidation immunotherapy after concurrent chemoradiotherapy | 57 | 132 | Asked only after endorsement of concurrent chemoradiotherapy in the unresectable branch |

**Table S2.** Branch Accounting for the Primary Outcome

| **Category** | **Count** | **% of possible respondent-vignette opportunities** | **Definition** |
| --- | --- | --- | --- |
| Observed neoadjuvant chemoimmunotherapy response | 283 | 60.3 | Eligible item was displayed and answered |
| Structurally inapplicable after unresectable judgement | 141 | 30.1 | Item was not displayed because the unresectable branch was entered |
| Vignette not reached | 42 | 9.0 | The vignette’s initial resectability item was not answered |
| True item non-response within resectable branch | 3 | 0.6 | Eligible item was displayed but left unanswered |

Structural inapplicability indicates that the item was not displayed because the respondent entered the opposite survey branch. Vignette not reached indicates that the initial resectability item for that vignette was not answered. True item non-response indicates that an eligible displayed item was left unanswered. Percentages are calculated using 469 possible respondent-vignette opportunities among the 67 respondents who answered the primary outcome at least once.

**Table S3.** Co-endorsement of Upfront Surgery and Neoadjuvant Chemoimmunotherapy

|  | **Neoadjuvant chemoimmunotherapy yes** | **Neoadjuvant chemoimmunotherapy no** | **Total** |
| --- | --- | --- | --- |
| Upfront surgery yes | 31 (11.1%) | 65 (23.2%) | 96 (34.3%) |
| Upfront surgery no | 182 (65.0%) | 2 (0.7%) | 184 (65.7%) |
| Total | 213 (76.1%) | 67 (23.9%) | 280 (100%) |

Restricted to resectable-branch respondent-vignette observations in which both items were answered. Treatment items were independent yes/no endorsements and were not mutually exclusive. This table should not be interpreted as a forced-choice treatment-allocation table.

**Table S4.** Karnofsky Performance Status Thresholds

| Vignette No. and label | Surgery / neoadjuvant chemoimmunotherapy | Concurrent chemoradiotherapy |
| --- | --- | --- |
| 1. T3 (>5 cm) N0 | 60 [50–70]; *n* = 65 | — |
| 2. T1/2 N1 (11L) | 60 [50–70]; *n* = 62 | — |
| 3. T1/2 N2 single station (4R) | 60 [50–70]; *n* = 55 | 50 [40–50]; *n* = 6 |
| 4. T1/2 N2 multi-station (4R+7) | 60 [50–80]; *n* = 43 | 60 [50–60]; *n* = 18 |
| 5. T3/4 N1 (11R) | 60 [50–70]; *n* = 46 | 50 [40–60]; *n* = 9 |
| 6. T3/4 N2 single station (4L) | 60 [60–70]; *n* = 7 | 60 [40–70]; *n* = 45 |
| 7. T3/4 N2 multi-station (2R+4R) | 60 [60–70]; *n* = 2 | 60 [50–70]; *n* = 48 |

Values are median [interquartile range], rounded to conventional 10-point categories.
