# Supplementary material for: Resectability and Neoadjuvant Chemoimmunotherapy in Stage II-III Non-Small Cell Lung Cancer: A European Case-Vignette Survey
Source: Interdiscip Cardiovasc Thorac Surg. 2026 Jul 7;41(7):ivag196. doi: 10.1093/icvts/ivag196 (PMC13385344; doi:10.1093/icvts/ivag196)
Supplement: ivag196_Supplementary_Data [file ivag196_supplementary_data.zip › Supplementary Material 1.pdf]

Perspectives on Resectability and Upfront Neoadjuvant Chemo-Immunotherapy in the UK and Europe

Dear Colleague,

Thank you for participating in our survey on "Perspectives on Resectability and Upfront Neoadjuvant Chemo-Immunotherapy in the UK and Europe."

This survey consists of seven clinical cases. For each case, please evaluate the scenario based on your professional experience and current clinical practices.

Please answer all questions for each case to the best of your ability. Your responses are anonymous and will be used solely for research purposes.

To take a closer look at any image included in the survey, right-click on the image and select "Magnify Image" to see it in full size.

We appreciate your time and contribution to this study.

Thank you!

In which country do you primarily practice?

____

What is your role?

- ◯ Thoracic Surgeon
- ◯ Clinical Oncologist
- ◯ Medical Oncologist
- ◯ Respiratory Physician
- ◯ Other (please specify)

Please Specify

____

CASE 1

> 5cm (T3) N0

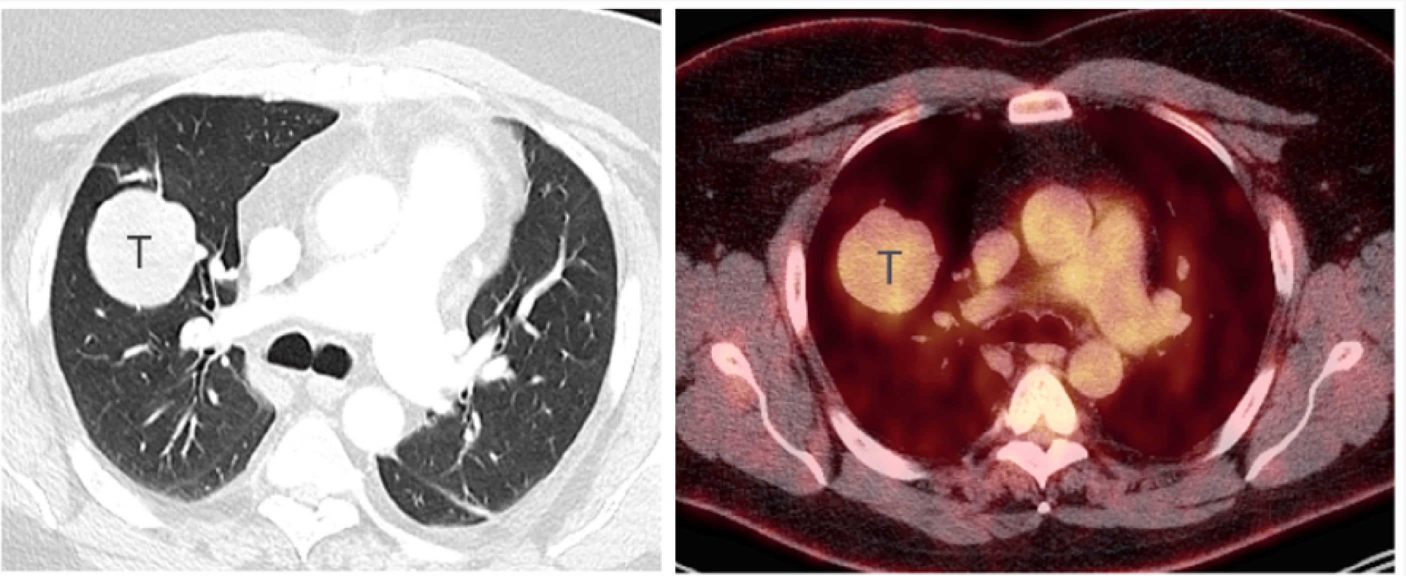

Is this resectable?	◯ Yes<br>◯ No
Would you recommend upfront resection?	◯ Yes<br>◯ No
If it was available to you, would you recommend neo-adjuvant chemo-immunotherapy first?	◯ Yes<br>◯ No

KARNOFSKY PERFORMANCE STATUS SCALE DEFINITIONS RATING (%) CRITERIA

Able to carry on normal activity and to work; no special care needed.	100	Normal no complaints; no evidence of disease.
	90	Able to carry on normal activity; minor signs or symptoms of disease.
	80	Normal activity with effort; some signs or symptoms of disease.
Unable to work; able to live at home and care for most personal needs; varying amount of assistance needed.	70	Cares for self; unable to carry on normal activity or to do active work.
	60	Requires occasional assistance, but is able to care for most of his personal needs.
	50	Requires considerable assistance and frequent medical care.
Unable to care for self; requires equivalent of institutional or hospital care; disease may be progressing rapidly.	40	Disable; requires special care and assistance.
	30	Severely disabled; hospital admission is indicated although death not imminent.
	20	Very sick; hospital admission necessary; active supportive treatment necessary.
	10	Moribund; fatal processes progressing rapidly.
	0	Dead

What is the Karnofsky performance status threshold below which you would not recommend surgery or induction treatment for this case?

____
(%)

If it was available to you, would you recommend perioperative immunotherapy (i.e. neoadjuvant AND adjuvant treatment)?

◯ Yes
◯ No

If the patient was fit, would you recommend concurrent chemo-radiation?

◯ Yes
◯ No

KARNOFSKY PERFORMANCE STATUS SCALE DEFINITIONS RATING (%) CRITERIA

Able to carry on normal activity and to work; no special care needed.	100	Normal no complaints; no evidence of disease.
	90	Able to carry on normal activity; minor signs or symptoms of disease.
	80	Normal activity with effort; some signs or symptoms of disease.
Unable to work; able to live at home and care for most personal needs; varying amount of assistance needed.	70	Cares for self; unable to carry on normal activity or to do active work.
	60	Requires occasional assistance, but is able to care for most of his personal needs.
	50	Requires considerable assistance and frequent medical care.
Unable to care for self; requires equivalent of institutional or hospital care; disease may be progressing rapidly.	40	Disable; requires special care and assistance.
	30	Severely disabled; hospital admission is indicated although death not imminent.
	20	Very sick; hospital admission necessary; active supportive treatment necessary.
	10	Moribund; fatal processes progressing rapidly.
	0	Dead

What is the Karnofsky performance status threshold below which you would not recommend concurrent chemo-radiation for this case?

____
(%)

If it was available to you, would you recommend adjuvant immunotherapy after concurrent chemo-radiation?

◯ Yes
◯ No

Why not?

____

CASE 2

T1/2 N1 (11L)

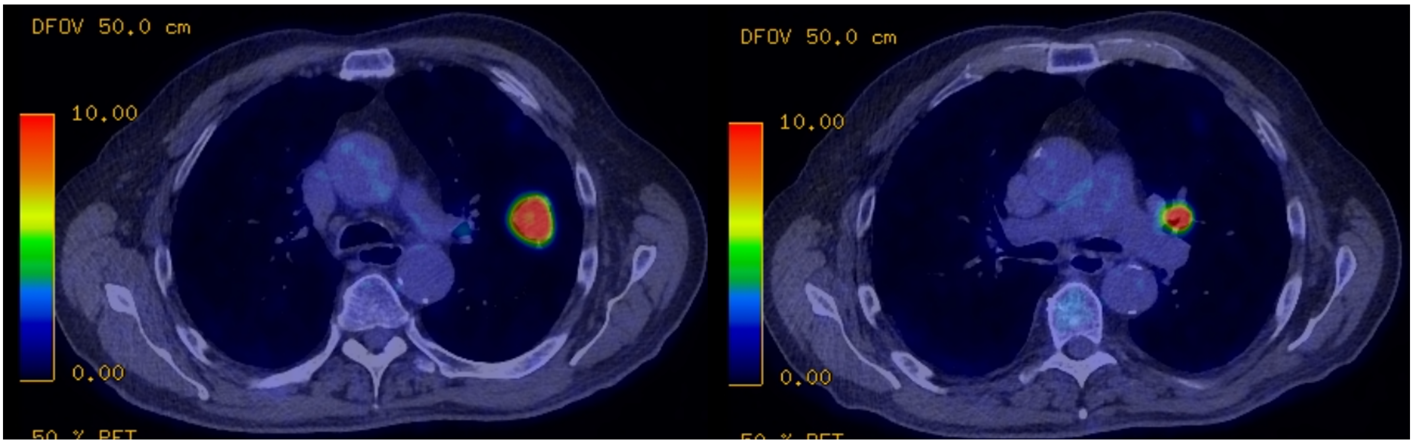

Is this resectable?	◯ Yes<br>◯ No
Would you recommend upfront resection?	◯ Yes<br>◯ No
If it was available to you, would you recommend neo-adjuvant chemo-immunotherapy first?	◯ Yes<br>◯ No

KARNOFSKY PERFORMANCE STATUS SCALE DEFINITIONS RATING (%) CRITERIA

Able to carry on normal activity and to work; no special care needed.	100	Normal no complaints; no evidence of disease.
	90	Able to carry on normal activity; minor signs or symptoms of disease.
	80	Normal activity with effort; some signs or symptoms of disease.
Unable to work; able to live at home and care for most personal needs; varying amount of assistance needed.	70	Cares for self; unable to carry on normal activity or to do active work.
	60	Requires occasional assistance, but is able to care for most of his personal needs.
	50	Requires considerable assistance and frequent medical care.
Unable to care for self; requires equivalent of institutional or hospital care; disease may be progressing rapidly.	40	Disable; requires special care and assistance.
	30	Severely disabled; hospital admission is indicated although death not imminent.
	20	Very sick; hospital admission necessary; active supportive treatment necessary.
	10	Moribund; fatal processes progressing rapidly.
	0	Dead

What is the Karnofsky performance status threshold below which you would not recommend surgery or induction treatment for this case? ______________________ (%)

If it was available to you, would you recommend perioperative immunotherapy (i.e. neoadjuvant AND adjuvant treatment)?

◯ Yes
◯ No

If the patient was fit, would you recommend concurrent chemo-radiation?

◯ Yes
◯ No

KARNOFSKY PERFORMANCE STATUS SCALE DEFINITIONS RATING (%) CRITERIA

Able to carry on normal activity and to work; no special care needed.	100	Normal no complaints; no evidence of disease.
	90	Able to carry on normal activity; minor signs or symptoms of disease.
	80	Normal activity with effort; some signs or symptoms of disease.
Unable to work; able to live at home and care for most personal needs; varying amount of assistance needed.	70	Cares for self; unable to carry on normal activity or to do active work.
	60	Requires occasional assistance, but is able to care for most of his personal needs.
	50	Requires considerable assistance and frequent medical care.
Unable to care for self; requires equivalent of institutional or hospital care; disease may be progressing rapidly.	40	Disable; requires special care and assistance.
	30	Severely disabled; hospital admission is indicated although death not imminent.
	20	Very sick; hospital admission necessary; active supportive treatment necessary.
	10	Moribund; fatal processes progressing rapidly.
	0	Dead

What is the Karnofsky performance status threshold below which you would not recommend concurrent chemo-radiation for this case?

____
(%)

If it was available to you, would you recommend adjuvant immunotherapy after concurrent chemo-radiation?

◯ Yes
◯ No

Why not?

____

CASE 3

T1/2 N2 Single Station (4R)

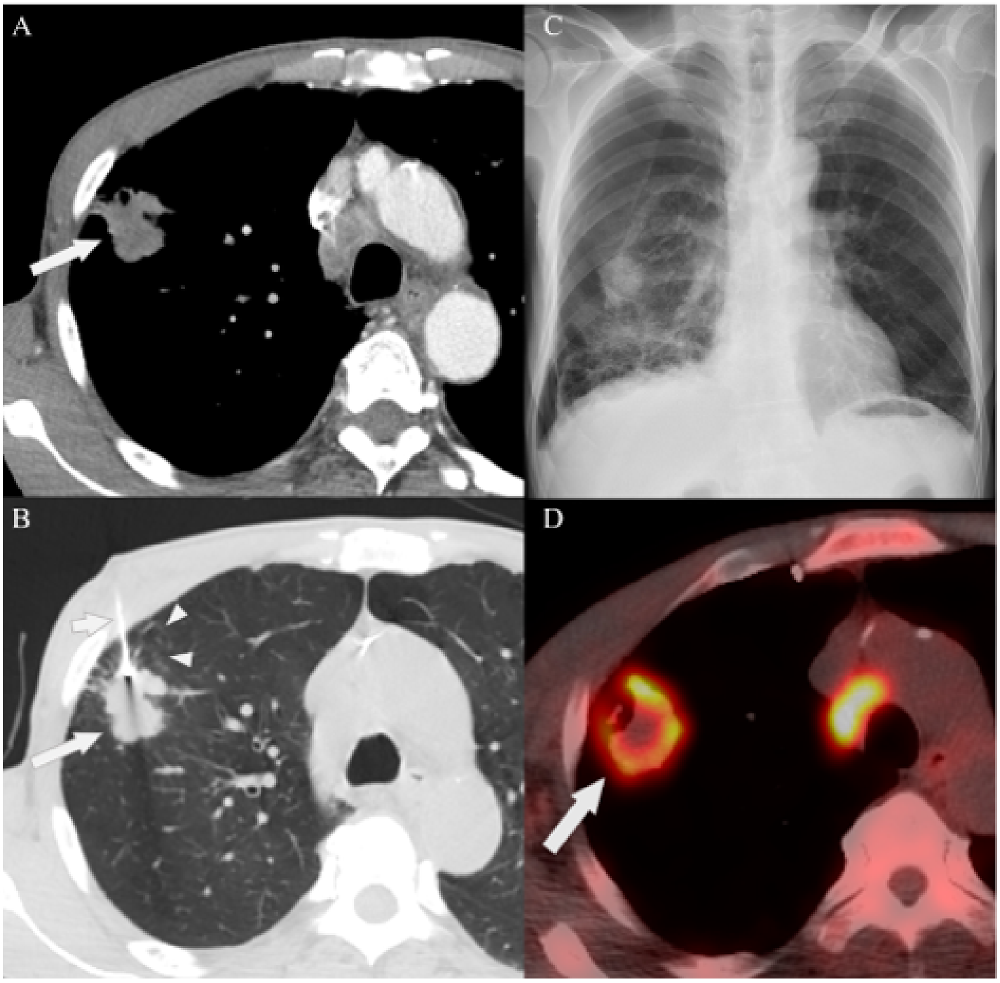

Is this resectable?	◯ Yes<br>◯ No
Would you recommend upfront resection?	◯ Yes<br>◯ No
If it was available to you, would you recommend neo-adjuvant chemo-immunotherapy first?	◯ Yes<br>◯ No

KARNOFSKY PERFORMANCE STATUS SCALE DEFINITIONS RATING (%) CRITERIA

Able to carry on normal activity and to work; no special care needed.	100	Normal no complaints; no evidence of disease.
	90	Able to carry on normal activity; minor signs or symptoms of disease.
	80	Normal activity with effort; some signs or symptoms of disease.
Unable to work; able to live at home and care for most personal needs; varying amount of assistance needed.	70	Cares for self; unable to carry on normal activity or to do active work.
	60	Requires occasional assistance, but is able to care for most of his personal needs.
	50	Requires considerable assistance and frequent medical care.
Unable to care for self; requires equivalent of institutional or hospital care; disease may be progressing rapidly.	40	Disable; requires special care and assistance.
	30	Severely disabled; hospital admission is indicated although death not imminent.
	20	Very sick; hospital admission necessary; active supportive treatment necessary.
	10	Moribund; fatal processes progressing rapidly.
	0	Dead

What is the Karnofsky performance status threshold below which you would not recommend surgery or induction treatment for this case?

____
(%)

If it was available to you, would you recommend perioperative immunotherapy (i.e. neoadjuvant AND adjuvant treatment)?

◯ Yes
◯ No

If the patient was fit, would you recommend concurrent chemo-radiation?

◯ Yes
◯ No

projectredcap.org

KARNOFSKY PERFORMANCE STATUS SCALE DEFINITIONS RATING (%) CRITERIA

Able to carry on normal activity and to work; no special care needed.	100	Normal no complaints; no evidence of disease.
	90	Able to carry on normal activity; minor signs or symptoms of disease.
	80	Normal activity with effort; some signs or symptoms of disease.
Unable to work; able to live at home and care for most personal needs; varying amount of assistance needed.	70	Cares for self; unable to carry on normal activity or to do active work.
	60	Requires occasional assistance, but is able to care for most of his personal needs.
	50	Requires considerable assistance and frequent medical care.
Unable to care for self; requires equivalent of institutional or hospital care; disease may be progressing rapidly.	40	Disable; requires special care and assistance.
	30	Severely disabled; hospital admission is indicated although death not imminent.
	20	Very sick; hospital admission necessary; active supportive treatment necessary.
	10	Moribund; fatal processes progressing rapidly.
	0	Dead

What is the Karnofsky performance status threshold below which you would not recommend concurrent chemo-radiation for this case?

____
(%)

If it was available to you, would you recommend adjuvant immunotherapy after concurrent chemo-radiation?

◯ Yes
◯ No

Why not?

____

CASE 4

T1/2 N2 Multi Station (4R and 7) Note: Station 7 slide not provided

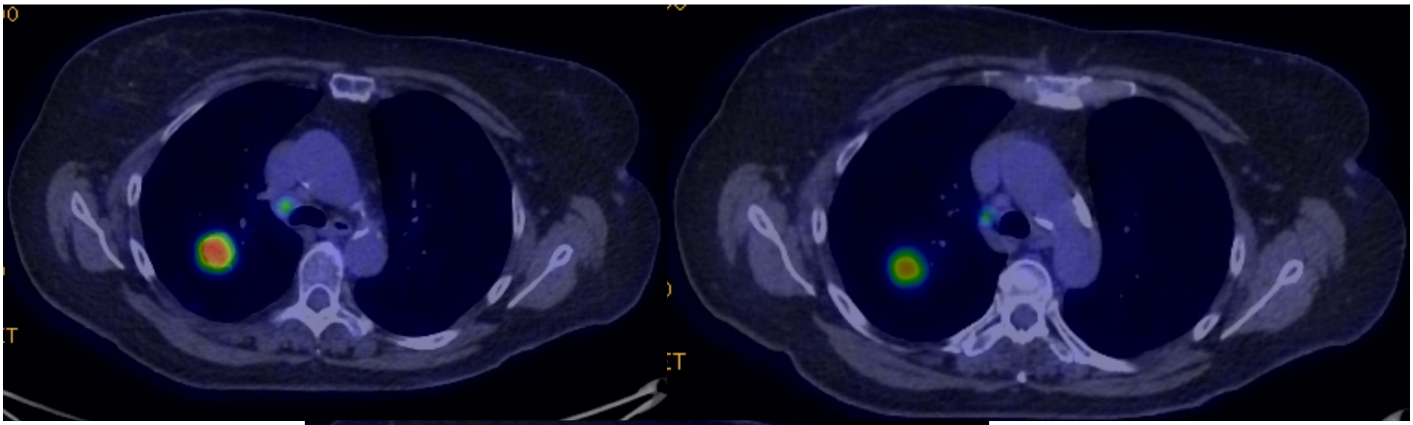

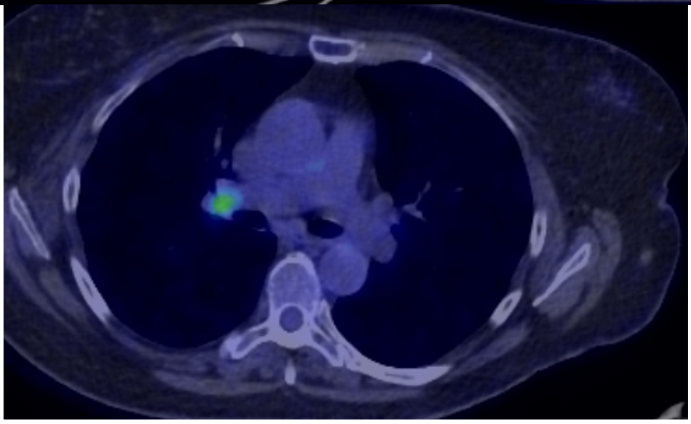

Is this resectable?	◯ Yes<br>◯ No
Would you recommend upfront resection?	◯ Yes<br>◯ No
If it was available to you, would you recommend neo-adjuvant chemo-immunotherapy first?	◯ Yes<br>◯ No

KARNOFSKY PERFORMANCE STATUS SCALE DEFINITIONS RATING (%) CRITERIA

Able to carry on normal activity and to work; no special care needed.	100	Normal no complaints; no evidence of disease.
	90	Able to carry on normal activity; minor signs or symptoms of disease.
	80	Normal activity with effort; some signs or symptoms of disease.
Unable to work; able to live at home and care for most personal needs; varying amount of assistance needed.	70	Cares for self; unable to carry on normal activity or to do active work.
	60	Requires occasional assistance, but is able to care for most of his personal needs.
	50	Requires considerable assistance and frequent medical care.
Unable to care for self; requires equivalent of institutional or hospital care; disease may be progressing rapidly.	40	Disable; requires special care and assistance.
	30	Severely disabled; hospital admission is indicated although death not imminent.
	20	Very sick; hospital admission necessary; active supportive treatment necessary.
	10	Moribund; fatal processes progressing rapidly.
	0	Dead

What is the Karnofsky performance status threshold below which you would not recommend surgery or induction treatment for this case?

____
(%)

If it was available to you, would you recommend perioperative immunotherapy (i.e. neoadjuvant AND adjuvant treatment)?

◯ Yes
◯ No

If the patient was fit, would you recommend concurrent chemo-radiation?

◯ Yes
◯ No

KARNOFSKY PERFORMANCE STATUS SCALE DEFINITIONS RATING (%) CRITERIA

Able to carry on normal activity and to work; no special care needed.	100	Normal no complaints; no evidence of disease.
	90	Able to carry on normal activity; minor signs or symptoms of disease.
	80	Normal activity with effort; some signs or symptoms of disease.
Unable to work; able to live at home and care for most personal needs; varying amount of assistance needed.	70	Cares for self; unable to carry on normal activity or to do active work.
	60	Requires occasional assistance, but is able to care for most of his personal needs.
	50	Requires considerable assistance and frequent medical care.
Unable to care for self; requires equivalent of institutional or hospital care; disease may be progressing rapidly.	40	Disable; requires special care and assistance.
	30	Severely disabled; hospital admission is indicated although death not imminent.
	20	Very sick; hospital admission necessary; active supportive treatment necessary.
	10	Moribund; fatal processes progressing rapidly.
	0	Dead

What is the Karnofsky performance status threshold below which you would not recommend concurrent chemo-radiation for this case?

____
(%)

If it was available to you, would you recommend adjuvant immunotherapy after concurrent chemo-radiation?

◯ Yes
◯ No

Why not?

____

projectredcap.org REDCap®

CASE 5

T3/4 N1 (11R)

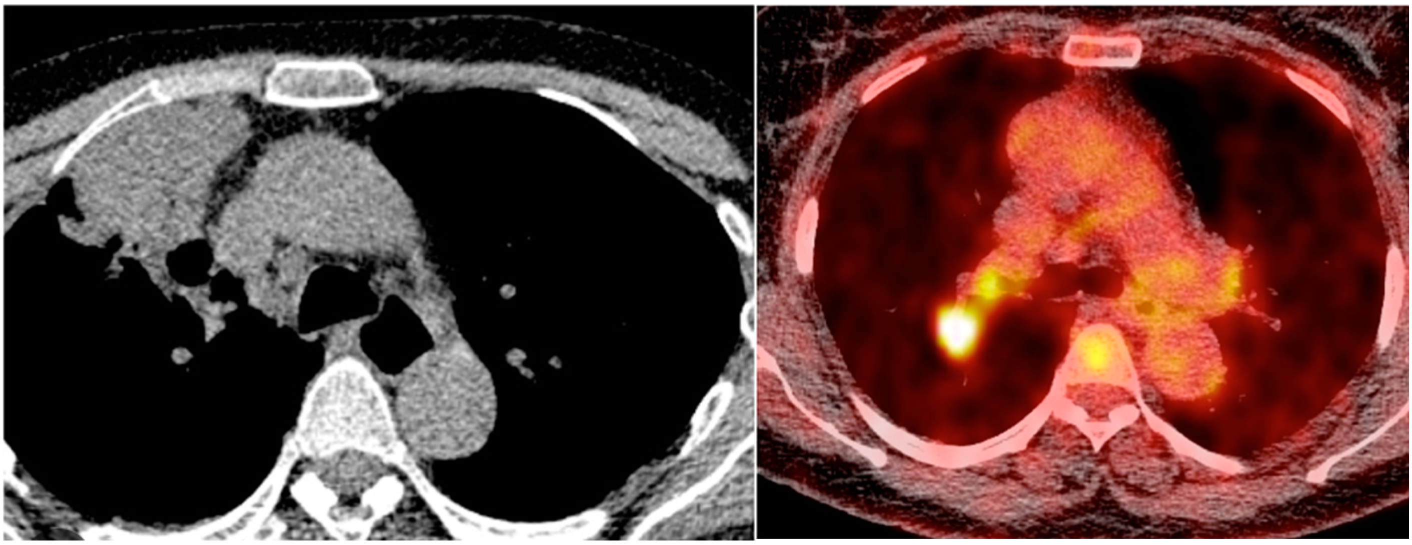

Is this resectable?	◯ Yes<br>◯ No
Would you recommend upfront resection?	◯ Yes<br>◯ No
If it was available to you, would you recommend neo-adjuvant chemo-immunotherapy first?	◯ Yes<br>◯ No

KARNOFSKY PERFORMANCE STATUS SCALE DEFINITIONS RATING (%) CRITERIA

Able to carry on normal activity and to work; no special care needed.	100	Normal no complaints; no evidence of disease.
	90	Able to carry on normal activity; minor signs or symptoms of disease.
	80	Normal activity with effort; some signs or symptoms of disease.
Unable to work; able to live at home and care for most personal needs; varying amount of assistance needed.	70	Cares for self; unable to carry on normal activity or to do active work.
	60	Requires occasional assistance, but is able to care for most of his personal needs.
	50	Requires considerable assistance and frequent medical care.
Unable to care for self; requires equivalent of institutional or hospital care; disease may be progressing rapidly.	40	Disable; requires special care and assistance.
	30	Severely disabled; hospital admission is indicated although death not imminent.
	20	Very sick; hospital admission necessary; active supportive treatment necessary.
	10	Moribund; fatal processes progressing rapidly.
	0	Dead

What is the Karnofsky performance status threshold below which you would not recommend surgery or induction treatment for this case?

__________________________________ (%)

If it was available to you, would you recommend perioperative immunotherapy (i.e. neoadjuvant AND adjuvant treatment)?

◯ Yes
◯ No

If the patient was fit, would you recommend concurrent chemo-radiation?

◯ Yes
◯ No

KARNOFSKY PERFORMANCE STATUS SCALE DEFINITIONS RATING (%) CRITERIA

Able to carry on normal activity and to work; no special care needed.	100	Normal no complaints; no evidence of disease.
	90	Able to carry on normal activity; minor signs or symptoms of disease.
	80	Normal activity with effort; some signs or symptoms of disease.
Unable to work; able to live at home and care for most personal needs; varying amount of assistance needed.	70	Cares for self; unable to carry on normal activity or to do active work.
	60	Requires occasional assistance, but is able to care for most of his personal needs.
	50	Requires considerable assistance and frequent medical care.
Unable to care for self; requires equivalent of institutional or hospital care; disease may be progressing rapidly.	40	Disable; requires special care and assistance.
	30	Severely disabled; hospital admission is indicated although death not imminent.
	20	Very sick; hospital admission necessary; active supportive treatment necessary.
	10	Moribund; fatal processes progressing rapidly.
	0	Dead

What is the Karnofsky performance status threshold below which you would not recommend concurrent chemo-radiation for this case?

__________________________________ (%)

If it was available to you, would you recommend adjuvant immunotherapy after concurrent chemo-radiation?

◯ Yes
◯ No

Why not?

____

CASE 6

T3/4 N2 Single Station (4L)

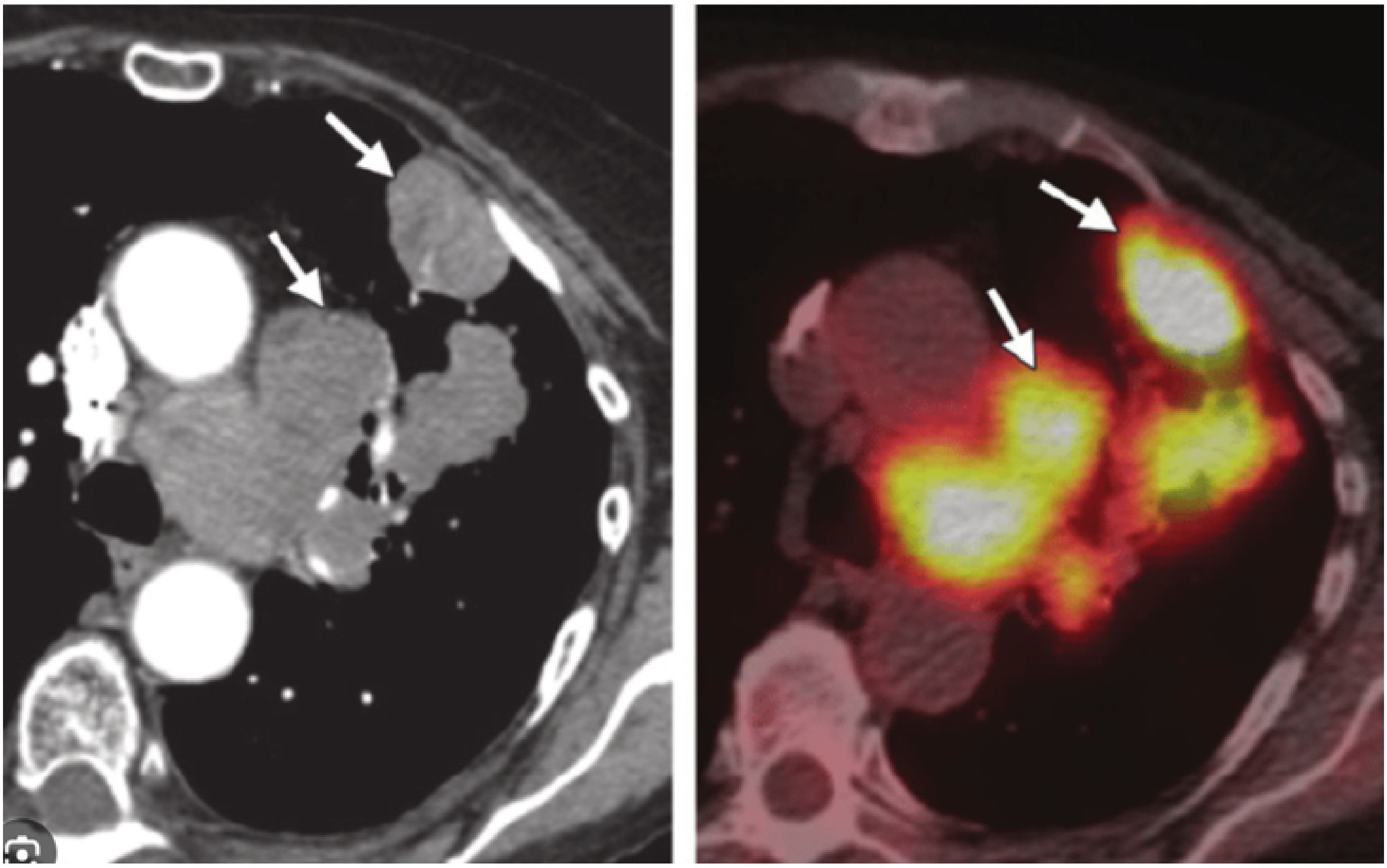

Is this resectable?	◯ Yes<br>◯ No
Would you recommend upfront resection?	◯ Yes<br>◯ No
If it was available to you, would you recommend neo-adjuvant chemo-immunotherapy first?	◯ Yes<br>◯ No

KARNOFSKY PERFORMANCE STATUS SCALE DEFINITIONS RATING (%) CRITERIA

Able to carry on normal activity and to work; no special care needed.	100	Normal no complaints; no evidence of disease.
	90	Able to carry on normal activity; minor signs or symptoms of disease.
	80	Normal activity with effort; some signs or symptoms of disease.
Unable to work; able to live at home and care for most personal needs; varying amount of assistance needed.	70	Cares for self; unable to carry on normal activity or to do active work.
	60	Requires occasional assistance, but is able to care for most of his personal needs.
	50	Requires considerable assistance and frequent medical care.
Unable to care for self; requires equivalent of institutional or hospital care; disease may be progressing rapidly.	40	Disable; requires special care and assistance.
	30	Severely disabled; hospital admission is indicated although death not imminent.
	20	Very sick; hospital admission necessary; active supportive treatment necessary.
	10	Moribund; fatal processes progressing rapidly.
	0	Dead

What is the Karnofsky performance status threshold below which you would not recommend surgery or induction treatment for this case?

____
(%)

If it was available to you, would you recommend perioperative immunotherapy (i.e. neoadjuvant AND adjuvant treatment)?

◯ Yes
◯ No

If the patient was fit, would you recommend concurrent chemo-radiation?

◯ Yes
◯ No

KARNOFSKY PERFORMANCE STATUS SCALE DEFINITIONS RATING (%) CRITERIA

Able to carry on normal activity and to work; no special care needed.	100	Normal no complaints; no evidence of disease.
	90	Able to carry on normal activity; minor signs or symptoms of disease.
	80	Normal activity with effort; some signs or symptoms of disease.
Unable to work; able to live at home and care for most personal needs; varying amount of assistance needed.	70	Cares for self; unable to carry on normal activity or to do active work.
	60	Requires occasional assistance, but is able to care for most of his personal needs.
	50	Requires considerable assistance and frequent medical care.
Unable to care for self; requires equivalent of institutional or hospital care; disease may be progressing rapidly.	40	Disable; requires special care and assistance.
	30	Severely disabled; hospital admission is indicated although death not imminent.
	20	Very sick; hospital admission necessary; active supportive treatment necessary.
	10	Moribund; fatal processes progressing rapidly.
	0	Dead

What is the Karnofsky performance status threshold below which you would not recommend concurrent chemo-radiation for this case?

______________________________ (%)

If it was available to you, would you recommend adjuvant immunotherapy after concurrent chemo-radiation?

◯ Yes
◯ No

Why not?

____

CASE 7

T3/4 N2 Multi Station (2R and 4R)

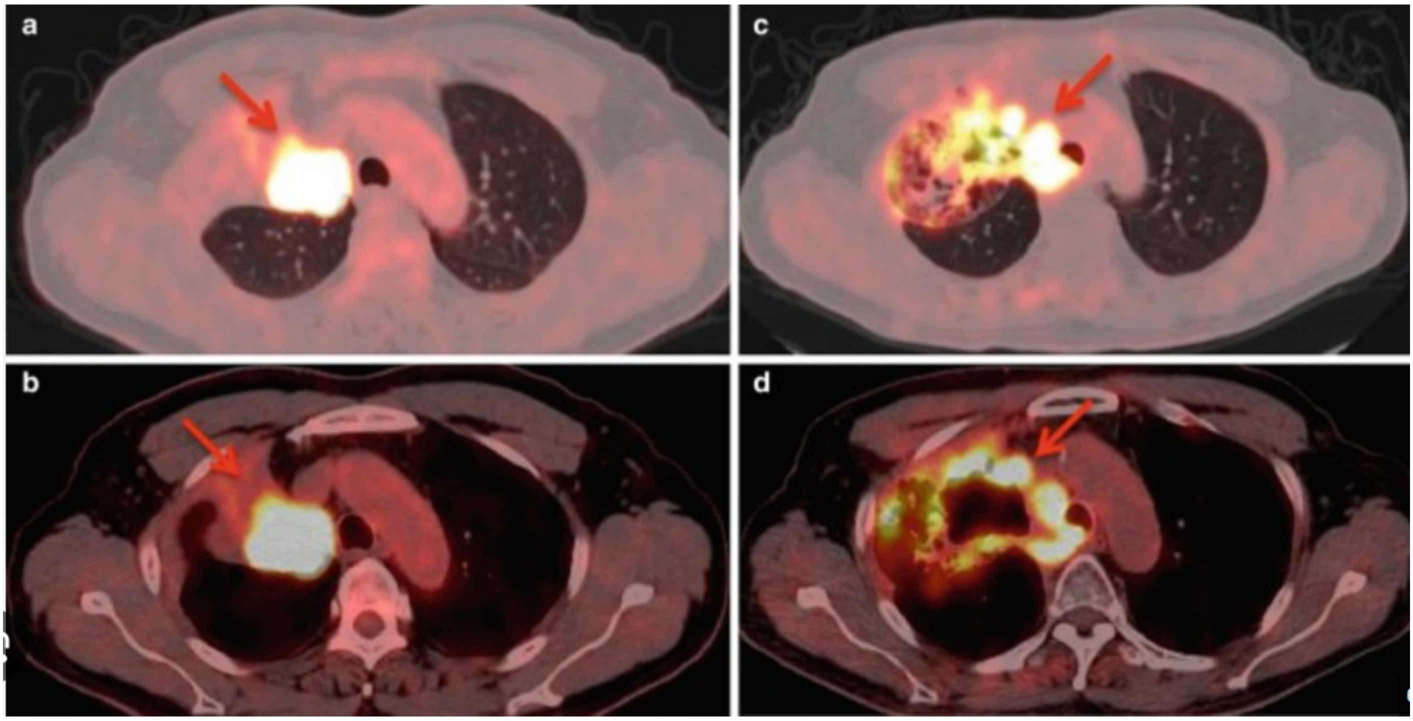

Is this resectable?	◯ Yes<br>◯ No
Would you recommend upfront resection?	◯ Yes<br>◯ No
If it was available to you, would you recommend neo-adjuvant chemo-immunotherapy first?	◯ Yes<br>◯ No

KARNOFSKY PERFORMANCE STATUS SCALE DEFINITIONS RATING (%) CRITERIA

Able to carry on normal activity and to work; no special care needed.	100	Normal no complaints; no evidence of disease.
	90	Able to carry on normal activity; minor signs or symptoms of disease.
	80	Normal activity with effort; some signs or symptoms of disease.
Unable to work; able to live at home and care for most personal needs; varying amount of assistance needed.	70	Cares for self; unable to carry on normal activity or to do active work.
	60	Requires occasional assistance, but is able to care for most of his personal needs.
	50	Requires considerable assistance and frequent medical care.
Unable to care for self; requires equivalent of institutional or hospital care; disease may be progressing rapidly.	40	Disable; requires special care and assistance.
	30	Severely disabled; hospital admission is indicated although death not imminent.
	20	Very sick; hospital admission necessary; active supportive treatment necessary.
	10	Moribund; fatal processes progressing rapidly.
	0	Dead

What is the Karnofsky performance status threshold below which you would not recommend surgery or induction treatment for this case?

________________________________ (%)

If it was available to you, would you recommend perioperative immunotherapy (i.e. neoadjuvant AND adjuvant treatment)?

◯ Yes
◯ No

If the patient was fit, would you recommend concurrent chemo-radiation?

◯ Yes
◯ No

KARNOFSKY PERFORMANCE STATUS SCALE DEFINITIONS RATING (%) CRITERIA

Able to carry on normal activity and to work; no special care needed.	100	Normal no complaints; no evidence of disease.
	90	Able to carry on normal activity; minor signs or symptoms of disease.
	80	Normal activity with effort; some signs or symptoms of disease.
Unable to work; able to live at home and care for most personal needs; varying amount of assistance needed.	70	Cares for self; unable to carry on normal activity or to do active work.
	60	Requires occasional assistance, but is able to care for most of his personal needs.
	50	Requires considerable assistance and frequent medical care.
Unable to care for self; requires equivalent of institutional or hospital care; disease may be progressing rapidly.	40	Disable; requires special care and assistance.
	30	Severely disabled; hospital admission is indicated although death not imminent.
	20	Very sick; hospital admission necessary; active supportive treatment necessary.
	10	Moribund; fatal processes progressing rapidly.
	0	Dead

What is the Karnofsky performance status threshold below which you would not recommend concurrent chemo-radiation for this case?

____
(%)

If it was available to you, would you recommend adjuvant immunotherapy after concurrent chemo-radiation?

◯ Yes
◯ No

Why not?

____

COMMENTS

Where did you hear about this survey?	◯ BTOG<br>◯ EACTS<br>◯ ESTS<br>◯ ERS<br>◯ ACP<br>◯ Other (please specify)
Please specify	____
Do you have any final comments, suggestions, or observations about the survey or the cases discussed?	____
